# Determinants of optimal antenatal care visit among pregnant women in Ethiopia: a multilevel analysis of Ethiopian mini demographic health survey 2019 data

**DOI:** 10.1186/s12978-022-01365-2

**Published:** 2022-03-05

**Authors:** Delelegn Emwodew Yehualashet, Binyam Tariku Seboka, Getanew Aschalew Tesfa, Tizalegn Tesfaye Mamo, Elias Seid

**Affiliations:** 1 School of Public Health, Dilla University, Dilla, Ethiopia; 2 College of Medicine and Health Science, Jimma University, Jimma, Ethiopia

**Keywords:** Optimal antenatal care visit, Determinants, Pregnant women, Ethiopia

## Abstract

**Background:**

Optimal antenatal care (ANC4+) needs to be used throughout pregnancy to reduce pregnancy complications and maternal mortality. The World Health Organization (WHO) recommends eight ANC contacts, while Ethiopia has the lowest coverage of at least four ANC visits. Therefore, this study aimed to identify factors associated with optimal ANC visits among pregnant women in Ethiopia.

**Methods:**

This study is a secondary data analysis of the 2019 Ethiopian Mini Demographic and Health Survey (EMDHS). A multilevel logistic regression model is set up to identify factors associated with optimal ANC visits. Adjusted odds ratios (AOR) with 95% confidence intervals (CI) were calculated to estimate the strength of the association between the outcome and the predictor variables.

**Results:**

Overall, 43% of women had optimal ANC visits during their last pregnancy. Higher educated women are 3.99 times more likely (AOR = 3.99; 95% CI: 2.62–6.02) to have optimal ANC visits than women with no formal education. The wealthiest women are 2.09 times more likely (AOR = 2.09; 95% CI: 1.56–2.82) to have optimal ANC visits than women in the poorest quintile. The odds of optimal ANC visit is 42 percent lower in rural women (AOR = 0.58, 95% CI: 0.41–0.83) compared to women living in urban areas.

**Conclusion:**

Women's educational status, wealth status, mass media exposure, place of residence and region are factors that are significantly associated with optimal ANC visit. These findings help health care programmers and policymakers to introduce appropriate policies and programs to ensure optimal ANC coverage. Priority should be given to addressing economic and educational interventions.

## Plain language summary

Optimal antenatal care means attending at least four antenatal care visits during pregnancy. In Ethiopia, evidence on factors affecting the use of at least four antenatal care services has not been adequately documented. Using the 2019 Ethiopian Mini Demographic and Health Survey data, this study attempted to uncover factors associated with optimal antenatal care visits among pregnant women in Ethiopia.

Data for women aged 15–49 who gave birth five years before the survey and attended antenatal care visits for their last pregnancy were taken from the 2019 Ethiopian Mini Demographic and Health Survey. Accordingly, 3927 women were included in the analysis.

Coverage of optimal antenatal care visits is low among pregnant women in Ethiopia. In this study, only 43% of pregnant women received optimal antenatal care. Women’s educational status, household wealth status, household size, mass media exposure, place of residence, and administrative region were associated with optimal antenatal care visits.

Strategies to increase access and availability of antenatal care services are important, especially for communities in rural areas and disadvantageous regions. Financial assistance that allows mothers from poor families to access antenatal care services can be beneficial. Health promotion programs targeting uneducated mothers are important to raise awareness of the importance of receiving a minimum of four antenatal care services.

## Introduction

Optimal antenatal care (ANC4+) is defined as attending at least four antenatal care (ANC) visits, injecting at least one dose of tetanus toxoid (TT), and consuming 100 iron-folic acids (IFA) tablets/syrup during pregnancy [1]. To ensure proper care, the WHO has previously recommended that every pregnant woman have at least four ANC visits throughout pregnancy, the first visit during the first trimester of pregnancy. However, in 2016, the WHO revised its recommended minimum number of ANC visits to four to eight contacts, with recent evidence showing that an increase in the number of contacts between a pregnant woman and a skilled health care provider can lead to reduced maternal mortality [1–4].

In 2017, nearly 295,000 women died during pregnancy and childbirth. The vast majority (94%) of these deaths occurred in low-income countries and were largely preventable [5, 6]. Sub-Saharan Africa (SSA) and South Asia accounted for about 86% (254,000) of global maternal deaths estimated in 2017 [6]. Recent statistics estimate that there are 534 maternal deaths per 100,000 live births in the SSA and 211 maternal deaths per 100,000 live births worldwide [7]. Ethiopia is one of the SSA countries with the highest maternal mortality rates in the world (412/100,000 live births) linked with low utilization of optimal ANC services and skilled delivery [8].

The use of at least four ANC visits during pregnancy promotes healthy practice that is proven to reduce maternal mortality and morbidity, as well as safe motherhood with improved maternal health outcomes [9, 10]. However, according to the 2018 Global Report, only three out of five (62%) women attended at least four ANC visits. In SSA, only 52% of women received at least four ANC visits [11].

In 2016, the WHO guideline recommended eight ANC visits to reduce pregnancy-related complications and maternal mortality, while four visits in Ethiopia are still lagging behind [1, 12]. The Ethiopian government in its Health Sector Transformation Plan (2015/16–2019/20) aims to reduce maternal mortality to 199/100000 live births and one of the strategies is achieving 95% ANC utilization of at least 4 visits. Despite several attempts by the government to achieve high coverage of four ANC visits, only 32% of pregnant women had 4 ANC visits [8]. A comprehensive understanding of the factors affecting low utilization is needed to inform policy and identify specific programming interventions so that greater coverage of optimal ANC visits and further improvements in maternal health care can be achieved.

Although research is underway to investigate the factors associated with the optimal use of ANC in Ethiopia, these studies are largely institutional-based and limited to specific areas and small sample sizes. There is a shortage of national data on factors associated with optimal ANC visits. Therefore, this study aimed to assess factors associated with optimal ANC visits among pregnant women in Ethiopia using nationally representative data.

## Methods

### Data source, study design, and setting

This study uses the latest Ethiopian Mini Demographic and Health Survey (EMDHS) 2019 data. The data was obtained from the DHS website (www.dhsprogram.com) after justifying the reason for the request. The 2019 EMDHS is a cross-sectional survey conducted in Ethiopia. Ethiopia is in the Horn of Africa. It covers a total area of ​​1,100,000 km2 and lies between latitude 3° and 15° north and longitude 33° and 48 east.

### Population and sampling procedure

The study population included all pregnant women five years before the survey. EMDHS 2019 uses a two-step stratified cluster sampling method in which sample households are selected in cluster enumeration areas (EAs). In the first stage, a total of 305 EAs were selected (93 in urban areas and 212 in rural areas) in proportion to EA size and with the independent selection at each sample level. In the second stage of selection, a fixed number of 30 households in each cluster are selected, with the possibility of systematically selecting the newly formed houses in the list. Any additional information related to the data collection, sample, and questionnaires used in the survey is detailed in 2019 EMDHS Report [12].

### Study variables

The outcome variable is optimal ANC visits, which is dichotomized as yes (if a woman has at least 4 ANC visits) and no (if a woman has less than 4 ANC visits). Both individual- and community-level factors are considered as potential predictor variables. Individual-level factors such as woman's age, women's educational status, wealth index, religion, household size, and mass media exposure. The two variables place of residence and region were considered as community-level factors.

### Data management and analysis

Data was extracted from EMDHS 2019 and further coding and analysis were done using STATA version 14.2. Sample weighting was done to adjust for the disproportionate allocation of samples to strata and regions in the survey process and to restore representation. Multivariable multilevel logistic regression is used to identify factors associated with optimal ANC visits at two levels: individual and community (cluster) levels. Four models have been set up for this multilevel logistic regression analysis. The first model is a blank model with no predictor variables to assess the extent of cluster variation on optimal ANC visits, the second model with individual-level variables, the third model with community-level variables, and the fourth model are with both individual- and community-level variables. A p-value of < 0.05 was used to define the statistical significance. Adjusted odds ratios (AORs) were calculated with their corresponding 95% confidence intervals (CIs) to determine independent predictors of optimal ANC visits. The inter-class correlation coefficient (ICC), a proportional change in variance (PCV), and the median odds ratio (MOR) were calculated to measure the variation between clusters. The Bayesian Deviance Information Criterion (DIC) was used to measure the fit of the models.

## Results

### Characteristics of the respondents

The analysis included data from a weighted sample of 3927 women aged 15–49 who gave birth five years before the survey and attended ANC visits for their last pregnancy. About 1409 (35.9%) women were between the ages of 20 and 34. The majority, 2900 (73.8%) of women are rural residents and 2014 (51.3%) of them have no formal education. Regarding household wealth status, 826 (21.0%) women belong to poor families. Overall, 43% of women had optimal ANC visits during their previous pregnancy. Only 37.4% of rural respondents had four or more ANC visits, while 58.7% of urban dwellers received four or more ANC visits. The proportion of optimal ANC visits was found to be higher among women in Addis Ababa (81.8%), highly educated women (79.0%), and women from the richest families (70.5%) (Table 1).

**Table 1:** Distribution of an optimal antenatal care visit by categories of selected variables among women’s in Ethiopian, EMDHS 2019

Variables	Count (%)	% of optimal ANC visit
Optimal ANC visit		
No	2238 (57.0)	
Yes	1688 (43.0)	100
Age at birth		
< 20	2506 (63.8)	39.7
20–34	1409 (35.9)	48.8
35–49	12 (0.3)	41.7
Education		
No education	2014 (51.3)	32.4
Primary	1415 (36.0)	47.0
Secondary	345 (8.8)	72.5
Higher	153 (3.9)	79.0
Wealth quintile		
Poorest	826 (21.0)	20.1
Poorer	822 (20.9)	37.7
Middle	761 (19.4)	39.0
Richer	705 (18.0)	48.8
Richest	813 (20.7)	70.5
Residence		
Urban	1027 (26.2)	58.7
Rural	2900 (73.8)	37.4
Region		
Tigray	287 (7.3)	63.8
Afar	51 (1.3)	31.4
Amhara	840 (21.4)	50.7
Oromia	1519 (38.7)	40.6
Somali	218 (5.6)	11.0
B/gumz	47 (1.2)	55.3
SNNPR	787 (20.0)	34.2
Gambela	19 (0.5)	31.6
Harari	11 (0.3)	36.4
Addis Ababa	127 (3.2)	81.8
Dire Dawa	21 (0.5)	61.9

### Random effects and model fitness

As shown in Table 2, the null model (Model 1) revealed a statistically significant variation in optimal ANC visits across communities (community variance = 1.9, p < 0.001), with 37.0% of the variation in the odds of optimal ANC visits is attributed for community-level factors (ICC = 37.0%). After adjusting the model for individual-level factors (Model 2), the variance in the odds of optimal ANC visit was statistically significant (community variance = 0.9, p < 0.001), with a variation of 21.2% in the odds of optimal ANC visits can be attributed to community-level factors.

**Table 2:** Random effect and model fitness showing the influence of community characteristics on optimal ANC visits in Ethiopia, EMDHS 2019

Parameter	Model 1	Model 2	Model 3	Model 4
Random effects				
Variance	1.9*	0.9*	0.7*	0.5*
PCV (%)	Reference	52.6	63.2	73.7
ICC (%)	37.0	21.2	16.9	12.5
MOR	3.7	2.5	2.2	1.9
Model fit statistics				
DIC (−2log likelihood)	4772	4498	4544	4364

Model 1—a model with no covariates; Model 2—only individual-level explanatory variables were included; Model 3—only community-level explanatory variables were included; Model 4 (Combined)—both individual and community level variables were included; *Significant at p < 0.001; *PCV* proportional change in variance, *ICC* intra-class correlation coefficient, *MOR* median odds ratio, *DIC* deviation information criteria

Adjusted for community-level factors, Model 3 revealed a statistically significant variation of optimal ANC visits in communities (community variance = 0.7, P < 0.001). In this model, 63.2% of community-level factors described variability in the optimal ANC visit (PCV = 63.2%), and 16.9% of the variation between clusters was attributed to community-level factors (ICC = 16.9%).

The final model (Model 4), which is adjusted for both individual- and community-level factors simultaneously, showed a statistically significant variance to the odds of optimal ANC visit (community variance = 0.5, P < 0.001). In this model, the variation between communities on optimal ANC visit was 12.5% due to community-level factors (ICC = 12.5%) and 73.7% of the variation in optimal ANC visit (PCV = 73.7%) was attributed to both individual and community-level factors.

Furthermore, the MOR for optimal ANC visit was 3.7 in the null model; this indicated that there is variation between communities (clustering) as the MOR is higher than the reference (MOR = 1). When all factors are added to the null model, the unexplained community variation in optimal ANC visit is reduced to MOR 1.9. This indicates that despite all factors being considered, the clustering effect is still statistically significant in the full model. In terms of model fitness, the final model (including both individual and community level factors) is the best-fitted model as it has the least deviance.

### Determinants of optimal ANC visit

Findings from multivariable multilevel analysis of individual and community-level factors are presented in Table 3. Individual factors that have a significant association with optimal ANC visits were women’s education status, household wealth status, household size, and mass media exposure, whereas, place of residence and region determine optimal ANC visits at the community level.

**Table 3:** Multivariable multilevel logistic regression analysis of factors associated with optimal ANC visit in Ethiopia, EMDHS 2019

Variables	Model 2 AOR (95% CI)	Model 3AOR (95% CI)	Model 4AOR (95% CI)
*Individual-level factors*			
Age			
< 20	1.00		1.00
20–34	1.13 (0.95–1.33)		1.09 (0.92–1.29)
35–49	0.53 (0.15–1.92)		0.44 (0.12–1.62)
Education			
No education	1.00		1.00
Primary	1.69 (1.39–2.05)		1.70 (1.41–2.06)***
Secondary	2.91 (2.12–3.98)		2.94 (2.15–4.03)^***^
Higher	4.02 (2.63–6.12)		3.99 (2.62–6.09)^***^
Wealth index			
Poorest	1.00		1.00
Poorer	1.85 (1.43–2.39)		1.63 (1.26–2.11)***
Middle	1.87 (1.41–2.47)		1.61 (1.21–2.12)**
Richer	2.41 (1.79–3.24)		2.09 (1.56–2.82)***
Richest	2.75(1.87–4.05)		1.78 (1.17–2.72)**
Having TV			
No	1.00		1.00
Yes	2.23 (1.59–3.12)		1.98 (1.41–2.79)^***^
Household size			
< 5	1.00		1.00
5–10	0.81 (0.67–0.98)		0.81 (0.67–0.99)^**^
> 10	0.36 (0.22–0.61)		0.37 (0.22–0.61)^***^
*Community-level factors*			
Residence			
Urban		1.00	1.00
Rural		0.27 (0.19–0.38)	0.58 (0.41–0.83)**
Region			
Addis Ababa		1.00	1.00
Tigray		0.89 (0.45–1.79)	1.19 (0.62–2.28)
Afar		0.16 (0.08–0.33)	0.39 (0.20–0.76)**
Amhara		0.59 (0.30–1.14)	0.89 (0.48–1.69)
Oromia		0.37 (0.19–0.72)	0.53 (0.29–0.99)*
Somali		0.03 (0.02–0.07)	0.09 (0.04–0.19)***
Benishangul Gumz		0.63 (0.31–1.26)	0.99 (0.52–1.92)
SNNPR		0.25 (0.13–0.48)	0.34 (0.18–0.65)**
Gambela		0.18 (0.09–0.36)	0.26(0.12–0.46)***
Harari		0.24 (0.12–0.47)	0.25 (0.14–0.47)***
Dire Dawa		0.55 (0.28–1.07)	0.74 (0.39–1.39)

*AOR* adjusted odds ratio, *CI* confidence interval, 1.00 = reference ^*^P < 0.05, ^**^P < 0.01, ^***^P < 0.001

Our results have proven that women’s educational status is an important positive determining factor for optimal ANC visits. Women with higher and secondary education were 3.99 times (AOR = 3.99; 95% CI: 2.62–6.02) and 2.94 times (AOR = 2.94; 95% CI: 2.15–4.03) more likely to have optimal ANC visits than women with no formal education. The richer women are 2.09 times more likely (AOR = 2.09; 95% CI: 1.56–2.82) to have the optimal ANC visits than women in the poorest quintile.

The administrative division has also been identified as an influencing factor of optimal ANC visits. The odds of optimal ANC visit is 42% lower in rural women (AOR = 0.58, 95% CI: 0.41–0.83) compared to women living in urban areas. Pregnant women living in the regional states of Ethiopia have lower odds of optimal ANC visits, Afar (AOR = 0.39, 95%CI: 0.20–0.76), Oromia (AOR = 0.53, 95%CI: 0.29–0.99), Somali (AOR = 0.09, 95%CI: 0.04–0.19), Southern Nations Nationalities and Peoples Region (SNNPR) (AOR = 0.34, 95%CI: 0.18–0.65), Gambela (AOR = 0.26, 95%CI: 0.12–0.46), Harari (AOR = 0.25, 95%CI: 0.14–0.47), compared to women in the capital city, Addis Ababa.

## Discussion

This study identified factors that are significantly associated with optimal ANC visits among pregnant women in Ethiopia. Our study found that 43% of women had optimal ANC visits during a previous pregnancy. It showed an improvement of 11% compared to the 2016 figures. This is due to the Government's efforts to make the community aware of the importance of ANC service in fulfilling the Millennium Development Goals (MDGs) through health sector development plans [13]. The average coverage rate of optimal ANC among 69 countries was approximately 55%, which is comparatively higher than Ethiopia [14]. Based on WHO recommendations, pregnant women should have at least eight ANC visits with a trained health care provider. This is especially necessary for developing countries as at least four ANC visits previously recommended are not sufficient to enhance the survival of mothers and children [1, 3]. However, our finding indicates that at least four ANC visits are still lagging in Ethiopia. Therefore, a more innovative approach should be introduced to improve the coverage of optimal ANC visits in Ethiopia.

According to findings from multi-country nationally representative data, the pooled prevalence of 8 or more ANC visits was 13% in the 15 studied countries [15]. Of the studied countries, Jordan had the highest prevalence of eight or more ANC contacts (74.0%), followed by Ghana (43.0%), and Albania (30.0%). In contrast, the prevalence of eight or more ANC contacts is very low in many countries, including Senegal, Uganda, Zambia, Mozambique, Mali, Guinea, Cameroon, and Benin. Many factors may have contributed to this low coverage of eight ANC visits. The 15 countries included in the study were low- and middle-income countries and among these poor-resource countries, health care services were inadequate, poor, or non-existent. Numerous studies show that maternal and child health care services are scarce in low- and middle-income countries, and that the majority of women in these poorer countries have no health insurance coverage [4, 16–21].

Moreover, evidence from Ghana showed that 41.9% of women had eight or more ANC contacts [22]. In contrast, the prevalence is lower in Nigeria (17.4%) [18] and the Republic of Benin (8.0%) [18]. The increase in Ghanaian women eight or more ANC contacts may be due to improved maternal and child health in Ghana in recent years [23]. As the policy seeks to completely eliminate the financial burden on pregnant women enrolled in Ghana's National Health Insurance Scheme (NHIS), many rural women and mothers from poorer and less privileged backgrounds are more likely to have access to maternal and child health care. This policy has provided helpful access to maternity health care in Ghana [24]. Therefore, to increase the coverage of a minimum of eight ANC contacts and access to maternity health care, policy formulation is needed on areas such as improved infrastructure, maternal and child health, women's empowerment, and comprehensive health insurance coverage, especially for women of reproductive age.

Women with higher education are more likely to have at least four ANC visits than uneducated women. This finding is consistent with previous studies in Ethiopia [25–27] and findings from the Republic of Benin [28], which showed that being educated or having a higher level of education had a positive influence on increasing the odds of having at least 4 ANC visits. This may be because educated women are more aware of the benefits of optimal ANC visits and have more understanding about pregnancy-related problems.

Our research indicated that household wealth status is an important determinant of optimal ANC visits. Women from the richest households had significantly more ANC visits than women from the poorest households. In line with this finding, studies from Bangladesh [29] and Indonesia [30] found that women from the wealthiest families were more likely to receive optimal ANC services than women from poorer families. This might be the improvement of the economy helps to advance health care utilization and able to afford medical and non-medical costs associated with ANC service during pregnancy [31–34]. Therefore, the results suggest that wealth status may be an important variable for determining optimal ANC visits. Low financial status means less money to transport to a health facility to use the ANC service. Another possible explanation is that women from wealthier families have proper education and more access to mass media than poor families.

Furthermore, the current study has shown that the likelihood of optimal ANC visits is 42% lower in rural women compared to women living in urban areas. Studies conducted in Nigeria [35] and Ethiopia [36] support this finding. This due to socio-economic inequalities and the gap in access to health services between urban and rural areas of the country [37]. Therefore, health insurance, free medical expenses, better human resources, construction of new health facilities, and road construction in rural areas may have contributed to the high coverage of optimal ANC visits in Ethiopia.

Our study also found a significant variation in optimal ANC visits between the administrative regions of Ethiopia. Women living in regional states are less likely to have optimal ANC visits than women living in Addis Ababa. This finding is supported by a previous study conducted in Ethiopia [38]. Both demand- and supply-side barriers, such as limited knowledge, transportation, and affordability issues, are responsible because ANC services cannot be accessed in more remote and difficult-to-reach areas. Addis Ababa is the capital city of the country, where health facilities are increasingly available and women are more aware of maternal health services.

### Strengths and limitations

Although previous studies have identified factors affecting the use of ANC services in Ethiopia, little is known about the determinants of having at least four ANC visits. It is in this context that the idea of this paper was developed to explore the contributing factors of optimal ANC visits in Ethiopia. The current study uses nationally representative survey data with large sample size. Therefore, it provide useful insights into the national-level determinants of optimal ANC visits in Ethiopia, which is highly informative to plan nationally appropriate strategies to achieve high coverage of optimal ANC contacts. As the current study uses recent survey data, it provides updated information on the predictors of having at least four ANC visits in Ethiopia. Another important strength of this study is the use of multilevel logistic regression analysis, which can detect factors other than individual-level factors that cannot be detected using standard logistic regression analysis. Despite the strengths of our study, we have some limitations. Since the data is of the cross-sectional type, it does not show causal inferences regarding individual and community level factors with optimal ANC visits. Furthermore, the use of secondary data prevented us from including some variables of interest that could have a significant influence on utilizing at least four ANC visits.

## Conclusion

The level of optimal ANC visits among women who gave birth within the last five years before the survey was low in Ethiopia. It was found that women’s educational status, household wealth status, household size, mass media exposure, place of residence, and region were significant determinants of optimal ANC visits. These findings help health care programmers and policymakers to introduce appropriate policies and programs to ensure optimal ANC coverage. Strategies to increase access and availability of antenatal care services are important, especially for communities in rural areas and disadvantageous regions. Since women with a poor family wealth index are less likely to attend optimal ANC visits in this study, financial support that allows mothers from poor families to afford costs associated with ANC services may be beneficial. Health campaigns targeting uneducated women are important to raise their awareness of the importance of attending a minimum of four antenatal care services.

## Data Availability

The data we used for this study are publicly available in the MEASURE DHS Program and you can access it from www.measuredhs.com once you have explained the objectives of the study. Upon receipt of the Authorization Letter, the data will be accessed and downloaded free.

## Abbreviations

ANC: Antenatal care
AOR: Adjusted odds ratio
CI: Confidence intervals
EMDHS: Ethiopian mini demographic health survey
ICC: Intra-cluster correlation
MOR: Median odds ratio
PCV: Proportional change in variance
SD: Standard deviation
WHO: World Health Organization

## References

[1] World Health Organization (2016). WHO Recommendations on Antenatal Care for a Positive Pregnancy Experience.

[2] Ataguba JE-O (2018). A reassessment of global antenatal care coverage for improving maternal health using sub-Saharan Africa as a case study. PLoS ONE.

[3] WHO Reproductive Health Library. WHO Recommendation on Antenatal Care Contact Schedules. WHO Reproductive Health Library Geneva World Health Organization; 2018.

[4] Ekholuenetale M, Nzoputam CI, Barrow A, Onikan A (2020). Women’s enlightenment and early antenatal care initiation are determining factors for the use of eight or more antenatal visits in Benin: further analysis of the demographic and health survey. J Egypt Public Health Assoc.

[5] WHO. Maternal health global situation 2020 2020. https:// www.who.int/health-topics/maternal-health#tab=tab_1.

[6] Trends in maternal mortality (2019). 2000 to 2017: estimates by WHO, UNICEF, UNFPA, World Bank Group and the United Nations Population Division.

[7] Okedo-Alex IN, Akamike IC, Ezeanosike OB, Uneke CJ. Determinants of antenatal care utilisation in sub-Saharan Africa: a systematic review. BMJ Open. 2019;9(10):e031890. https://bmjopen.bmj.com/content/9/10/e031890. 10.1136/bmjopen-2019-031890 PMC6797296 31594900

[8] Central Statistical Agency (CSA) and ICF, Ethiopia Demographic and Health Survey, Central Statistical Agency, Addis Ababa, Ethiopia, 2016.

[9] Kuhnt J, Vollmer S (2017). Antenatal care services and its implications for vital and health outcomes of children: evidence from 193 surveys in 69 low-income and middle-income countries. BMJ Open.

[10] Haftu A, Hagos H, Mehari M-A (2018). Pregnant women adherence level to antenatal care visit and its effect on perinatal outcome among mothers in Tigray public health institutions, 2017: cohort study. BMC Res Notes.

[11] UNICEF. Monitoring the situation of children and women. 2018.

[12] Ethiopian Public Health Institute (EPHI) [Ethiopia] and ICF. 2021. Ethiopia Mini Demographic and Health Survey 2019: Final Report. Rockville, Maryland, USA: EPHI and ICF.

[13] USAID, A. Three successful sub-Saharan Africa family planning programs: lessons for meeting the MDGs. Washington DC: USAID; 2012.

[14] Requejo JH, Bryce J, Barros AJ (2015). Countdown to 2015 and beyond: fulfilling the health agenda for women and children. Lancet.

[15] Ekholuenetale M (2021). Prevalence of eight or more antenatal care contacts: findings from multi-country nationally representative data. Glob Pediatr Health.

[16] Meda IB, Baguiya A, Ridde V, Ouédraogo HG, Dumont A, Kouanda S (2019). Out-of-pocket payments in the context of a free maternal health care policy in Burkina Faso: a national cross-sectional survey. Health Econ Rev.

[17] Ogundele OJ, Pavlova M, Groot W (2020). Socioeconomic inequalities in reproductive health care services across Sub-Saharan Africa. A systematic review and meta-analysis. Sex Reprod Healthc..

[18] Ekholuenetale M, Benebo FO, Idebolo AF (2020). Individual-, household-, and community-level factors associated with eight or more antenatal care contacts in Nigeria: evidence from demographic and health survey. PLoS ONE.

[19] Novignon J, Ofori B, Tabiri KG, Pulok MH (2019). Socioeconomic inequalities in maternal health care utilization in Ghana. Int J Equity Health.

[20] Nwosu CO, Ataguba JE (2019). Socioeconomic inequalities in maternal health service utilisation: a case of antenatal care in Nigeria using a decomposition approach. BMC Public Health.

[21] Pulok MH, Uddin J, Enemark U, Hossin MZ (2018). Socioeconomic inequality in maternal healthcare: an analysis of regional variation in Bangladesh. Health Place.

[22] Ekholuenetale M, Nzoputam CI, Barrow A (2021). Prevalence and socioeconomic inequalities in eight or more antenatal care contacts in Ghana: findings from 2019 population-based data. Int J Womens Health.

[23] Knoema. Ghana maternal mortality ratio, 1960–2020—knoema.com; 2021. https://knoema.com//atlas/Ghana/Maternal-mortality-ratio.

[24] Wang W, Temsah G, Mallick L (2016). The impact of health insurance on maternal health care utilization: evidence from Ghana Indonesia and Rwanda. Health Policy Plan.

[25] Basha GW (2019). Factors affecting the utilization of a minimum of four antenatal care services in Ethiopia. Obstet Gynecol Int.

[26] Muchie KF (2017). Quality of antenatal care services and completion of four or more antenatal care visits in Ethiopia: a finding based on a demographic and health survey. BMC Pregnancy Childbirth.

[27] Ousman SK, Mdala I, Thorsen VC, Sundby J, Magnus JH (2019). Social determinants of antenatal care service use in Ethiopia: changes over a 15-year span. Front Public Health.

[28] Dansou J, Adekunle AO, Arowojolu AO (2017). Factors associated with antenatal care services utilisation patterns amongst reproductive age women in Benin Republic: an analysis of 2011/2012 Benin Republic’s demographic and health survey data. Niger Postgrad Med J.

[29] Rahman A (2017). Trends, determinants and inequities of 4(+) ANC utilisation in Bangladesh. J Health Popul Nutr.

[30] Denny HM (2022). The determinants of four or more antenatal care visits among working women in Indonesia. Asia Pac J Public Health.

[31] Ataguba JE-O (2018). A reassessment of global antenatal care coverage for improving maternal health using sub-Saharan Africa as a case study. PLoSOne..

[32] Catherine Arsenault KJ, Lee D, Dinsa G, Manzi F, Marchant T, Kruk ME (2018). Equity in antenatal care quality: an analysis of 91 national household surveys. Lancet Glob Health.

[33] Gebreyohannes Y (2017). Improving antenatal care services utilization in Ethiopia: an evidence-based policy brief. Int J Health Econ Policy.

[34] Makate M, Makate C (2017). The evolution of socioeconomic status-related inequalities in maternal health care utilization: evidence from Zimbabwe, 1994–2011. Glob Health Res Policy.

[35] Adewuyi EO, Auta A, Khanal V, Bamidele OD, Akuoko CP, Adefemi K (2018). Prevalence and factors associated with underutilization of antenatal care services in Nigeria: a comparative study of rural and urban residences based on the 2013 Nigeria demographic and health survey. PLoS ONE.

[36] Tiruaynet K, Muchie KF (2019). Determinants of utilization of antenatal care services in Benishangul Gumuz region, Western Ethiopia: a study based on demographic and health survey. BMC Pregnancy Childbirth.

[37] Gebre E, Worku A, Bukola F (2018). Inequities in maternal health services utilization in Ethiopia 2000–2016: magnitude, trends, and determinants. Reprod Health.

[38] Yeneneh A, Alemu K, Dadi AF, Alamirrew A (2018). Spatial distribution of antenatal care utilization and associated factors in Ethiopia: evidence from Ethiopian demographic health surveys. BMC Pregnancy Childbirth.

